# A qualitative method for analysing multivoicedness

**DOI:** 10.1177/1468794114557991

**Published:** 2015-12

**Authors:** Emma-Louise Aveling, Alex Gillespie, Flora Cornish

**Affiliations:** University of Leicester, UK; London School of Economics and Political Science, UK; London School of Economics and Political Science, UK

**Keywords:** dialogical Self, dialogism, I-positions, method of analysis, multivoicedness, Other, voices

## Abstract

‘Multivoicedness’ and the ‘multivoiced Self’ have become important theoretical concepts guiding research. Drawing on the tradition of dialogism, the Self is conceptualised as being constituted by a multiplicity of dynamic, interacting voices. Despite the growth in literature and empirical research, there remains a paucity of established methodological tools for analysing the multivoiced Self using qualitative data. In this article, we set out a systematic, practical ‘how-to’ guide for analysing multivoicedness. Using theoretically derived tools, our three-step method comprises: identifying the voices of I-positions within the Self’s talk (or text), identifying the voices of ‘inner-Others’, and examining the dialogue and relationships between the different voices. We elaborate each step and illustrate our method using examples from a published paper in which data were analysed using this method. We conclude by offering more general principles for the use of the method and discussing potential applications.

## Introduction

Research on selfhood increasingly conceptualises ‘the Self’ as plural, multiple, or multivoiced, rather than as single or unitary (Hermans and Thorsten, 2011; Linell, 2009; Grossen and Salazar-Orvig, 2011). As William James (1890) observed, people have as many ‘selves’ as there are people or groups with whom they interact. Equally, for Bakhtin, Self arises in and through social relations with Others; Others are, in this sense, part of Self (Bakhtin, 1981: 354; Wertsch, 1991). The Self is always infused with and responding to the voices of Others. For example, people are concerned with what other people think and say, and people often repeat or paraphrase the words of others (Marková, 2003; Gillespie and Cornish, 2010). That is to say, the Self often thinks and speaks with the words of Others. Even when the Self speaks directly, the utterances often imply an Other because they are addressed to and anticipate the response of that Other (Grossen, 2010). This multivoiced nature of the Self has been considered an adaptive response to the fractured social world which we traverse (Aveling and Gillespie, 2008; Gioia, Schultz and Corley, 2000; Kerr, Crowe and Oades, 2013). But how can such multivoicedness be made apparent in empirical data? What techniques can researchers use to empirically analyse the multivoiced Self?

Despite a growing literature and many theoretical advances (Märtsin et al., 2011), there remains a paucity of established methodological tools for analysing multivoicedness (Gillespie and Cornish, in press). The aim of the present article is to contribute to the field of qualitative inquiry a systematic methodological approach for extracting the voices of Self and Other within talk or text, and analysing the relations between them. We begin by introducing existing literature on analytical approaches to the multivoiced Self; we then unpack the key theoretical concepts that underpin the method we present. The main body of the article presents a three-step method, which we call the ‘analysis of multivoicedness,’ and a worked example of the application of the method. We conclude by discussing principles for the use of the method and its potential applications.

### Empirical research on multivoicedness

Multivoicedness and the multivoiced Self have become important guiding concepts for research in a wide range of disciplines and fields (Cooper et al., 2012). This trend reflects paradigmatic shifts in the social and psychological sciences away from individualistic and mechanistic epistemologies, toward more dynamic, social alternatives that recognise the situated and intersubjective nature of meaning-making (Gillespie and Cornish, 2010). Hence a need has developed within empirical research for analytic tools which take as their fundamental unit of analysis the individual in interaction with others and his/her cultural, historical and institutional setting (Linell, 2009; Marková, 2003). We briefly review examples from the fields of intercultural contact, healthcare and education that apply the concepts of multivoicedness and the multivoiced Self.

Research on intercultural contact often conceptualises identities as ‘hybrid’ or ‘hyphenated’. Accordingly, researchers of multicultural selves have used the concept of multivoicedness to analyse that ‘hybridity’. For example, Bhatia’s (2002) analysis reveals how migrant and diasporic communities invoke the voices of host *and* home communities to position themselves within different social contexts. In a multicultural, urban American school context, Luttrell (2010) shows how participants’ photography and video-recordings engage with, appropriate and orient to multiple voices (present and absent in their daily lives) in the process of forming and claiming identities.

Research in healthcare communication has long focused on the interaction between health professional and patient. Analyses of multivoicedness have shown how the voices of professionals and patients are not completely distinct, but inter-penetrate. For instance, Roberts and Sarangi (1999) analyse the conversations in oral medical examinations, finding them to be ‘hybrid’ combinations of personal, professional and institutional discourses. Grossen and Salazar-Orvig (2011) examine psychotherapeutic consultations, exploring how the parties invoke the voices of people who are not present, such as teachers and doctors, as allies or counter-positions in their argumentation.

Finally, in the field of education, questions about the power of different voices have attracted particular interest. Research has examined the authority of the teacher’s voice in relation to students’ voices (Mortimer, 1998; Wertsch, 2004), and the power of different groups’ voices to influence the outcomes of collaborative projects (Akkerman et al., 2006). The voice of significant others has also been shown to have a powerful role in legitimating and valuing knowledge (Grossen, Zittoun and Ros, 2012).

Each of the above studies demonstrates that an insightful analysis of the voices within qualitative data is possible. Their analytic methods, however, have not been elaborated in any detail. Some studies use data which are (superficially) ‘dialogical’ in that they involve observations or recordings of people in interaction (e.g. Akkerman et al., 2006; Atkinson and DePalma, 2008; Luttrell, 2010). Gathering interaction data, however, is not sufficient to guarantee a dialogical analysis, as even such data may be analysed in ways which reduce and individualise rather than treating the interdependence of Self and Other as the basic, irreducible unit of analysis (Akkerman and Niessen, 2011). Equally, multivoicedness can be analysed within apparently non-interactional data, as in, for example, Bhatia’s (2002) analysis of autobiographical text. With Marková (2011), we suggest that the extent to which data is multivoiced (or ‘monological’) depends not on the method used to collect it but on the way the data is conceptualised and analysed. However, no systematic bridge from conceptualisation to analysis exists, which is a barrier to both the quantity and quality of future dialogical analyses.

The main attempts to formalise a methodology for studying the multivoiced Self have been in the field of psychology and have relied on self-reflection and self-report questionnaires (see Jasper et al., 2011 for a review). These approaches stem from ‘dialogical self theory’ (Hermans, 1999; Hermans and Dimaggio, 2007), in which the Self is conceptualised as a dynamic multiplicity of ‘I-positions’ from which the Self can speak and act. Methodologies such as the Personal Position Repertoire method (Hermans, 2001) map out this landscape of I-positions and their interactions (Jasper et al., 2011; Kluger, Nir and Kluger, 2008; Puchalska-Wasyl, Chemielnicka-Kuter and Oleś, 2008; Raggatt, 2000).

While these self-report questionnaires have advantages in terms of rigour and reliability, they also have two major limitations. First, the Personal Position Repertoire turns the voice into something that participants speak *about*, rather than a position they speak *from*. Participants may not be aware of all the voices with which they speak, and self-reports may reflect a limited view of Self’s multiplicity of voices; they may be disproportionately shaped by voices aligned with a socially desirable Self (Jasper et al., 2011). Second, data collection using these methods is usually done outside a natural context. Thus, use of these methods risks de-contextualising the voices and failing to analyse them in terms of situated social relations (Grossen, 2010).

Our contribution in the present article is to provide a set of explicit methodological steps for analysis of multivoicedness within qualitative data which takes account of context.

### Key concepts for analysing multivoicedness

Conceptualising the Self as multivoiced originates in the theoretical tradition of dialogism. Within this tradition, the Other is not in opposition to Self, but part of Self (Bakhtin, 1981: 354; Wertsch, 1991). Moreover, the Self is not simply what is self-reported, but also how the Self relates to Others (Marková, 2003). This emphasis on Self-in-relation-to-Others implies three foci for our method: voices of the Self, voices of Others, and their interactions. In this section we elaborate key theoretical concepts that allow us to unpack these voices and their interrelations as they appear within the multivoiced Self. While these theoretical concepts are rooted in the interdisciplinary tradition of dialogism, they have been further developed in multiple fields including linguistics (e.g. Linell, 2009), psychology (e.g. Hermans, 2001; Marková, 2003) and sociocultural research (Werstch, 1991).

#### 1) Voices of the Self: I-positions

There are two types of voices within the Self. First, and most obviously, there are the positions from which Self speaks – the ‘I’ in James’s (1890) terms. The ‘I’ can speak (and act) from a multiplicity of different ‘I-positions’ (Hermans, 2001). While each I-position is initially cultivated in a particular set of social relations and particular context, in a person’s psychological life, I-positions from various contexts collide, and within one context or even one utterance the Self may move between I-positions or voices. For example, a mother might speak as a mother, a woman, or someone who loves gardening.

#### 2) Voices of Other: inner-Others

The second type of voice within the Self comprises voices which are attributed to Others (but are distinct from the voices of actual Others). These voices of the Other within Self we call ‘inner-Others’ (Bakhtin, 1981; Marková, 2006). Inner-Others do not only represent ‘real’ individuals (e.g. my mother, my boss); they may also be imagined Others or generalised Others (e.g. my community) or reflect discourses or social languages associated with particular groups or institutions. Even those people (or groups) who the Self sees as radically ‘other’ (e.g. out-groups and even enemies) are inner-Others and thus part of the Self. They are positions in opposition to which the Self defines itself.

Voices of inner-Others may appear within the talk of the Self in at least three forms (Gillespie, 2006): in the form of direct quotes, where the speaker gives voice to a specific person or group (e.g. ‘my mother said xyz’); in the form of indirect quotes, where the speaker refers to the opinions, beliefs, utterances or ideas of another person or group (e.g. ‘they believe that people should xyz’); and in the form of ‘echoes’. Echoes pertain to a more subtle level of dialogicality, namely, the way in which most ideas and utterances are second-hand or borrowed. The words we use are always ‘half someone else’s’ (Bakhtin, 1981: 293–4), bearing traces of their former uses. Bakhtin (1986) used the term ‘ventriloquation’ to describe the situation of a speaker adopting an established social language, without fully owning it (Wertsch, 1991), that is, without it becoming an I-position. For example, a student might struggle to speak through a newly appropriated academic discourse. In such a case the attentive listener will hear ‘echoes’ of textbooks, teachers and perhaps other students. Echoes, then, are akin to unreferenced quotations.

#### 3) Interacting voices: heterodialogue and autodialogue

The third aspect concerns how the voices of Self and Other, I-positions and inner-Others, interact. Alongside dialogue with actual Others (which we will call ‘heterodialogue’) there can be instances of ‘autodialogue’ – dialogue between the voices within the Self (Josephs and Valsiner, 1998). This occurs, for example, when someone asks herself a question or when she interrupts herself to disagree with her own utterance or to quote someone else’s response. Inner-Others play an active role in autodialogue, changing topics, introducing ideas and shifting the positions from which individuals speak (Marková, 2006).

Like heterodialogue, however, autodialogue never takes place on neutral ground. Reflecting the sociocultural context from which the voices within the Self originate, the dialogical dynamics within the Self are characterised by patterns of dominance and asymmetrical power relations. Just as certain discourses or dominant groups may marginalise or suppress the voice of less powerful others, so too may certain voices within the Self dominate, undermine or silence others (Valsiner, 2002).

These theoretically-derived concepts can be operationalised as concrete tools for analysing multivoicedness empirically. In the next section we provide a practical, step-by-step method for applying these tools.

## Analysis of multivoicedness

**Figure 1.**
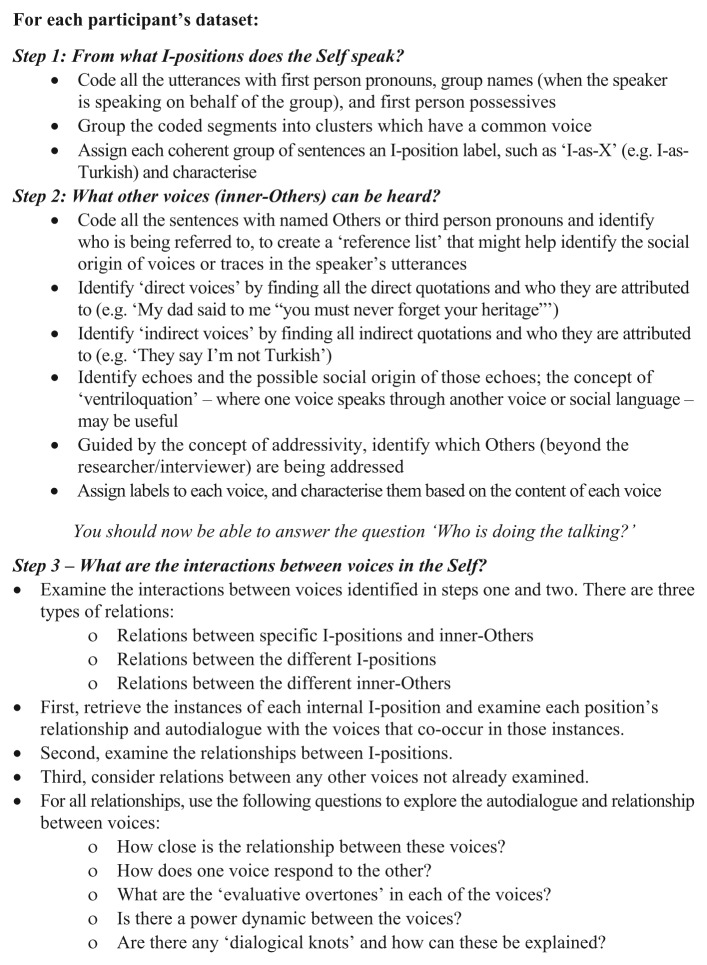
Summary of the steps in an analysis of multivoicedness.

The three-step method we present focuses on the voices of Self and inner-Others within Self’s discourse, and the interactions between these voices as they appear *within* the talk (or text) of individuals. Steps one and two address the question ‘who is doing the talking?’ by identifying, first, the multiplicity of I-positions from which Self speaks, and second, the voices of inner-Others that can be heard within the speaker’s utterances. The third step then examines the nature of the autodialogue and relationships between voices within the Self. The three steps are described in Figure 1. We illustrate each step using extracts from a published study of multivoicedness.

The steps can be applied to any textual data, but for the sake of simplicity, here we describe these steps in the context of analysing interview data. The process should be sequentially applied to individual transcripts. For practical purposes the three steps are presented categorically and in linear fashion. In practice, the analysis will require an iterative approach.

### Illustration: Second-generation Turkish immigrants in London

The data used for our illustration come from a qualitative study of identity construction amongst second-generation Turkish adolescents living in London (Aveling and Gillespie, 2008). We used the concept of the multivoiced Self to understand how the young people’s identity construction was related to and shaped by their sociocultural context.

Ten adolescents of a Turkish supplementary school in London participated in individual interviews and then in one of two focus groups. All had Turkish or Turkish Cypriot parents and had grown up in England. Interviews explored students’ accounts of themselves, family, friends, education, the Turkish community and their future aspirations, while focus groups explored common themes and contradictions. To support the analysis of multivoicedness, we utilised ethnographic and secondary data, including interviews with teachers, observations of school activities, personal websites and sociological literature on Turkish and Cypriot communities in England (Enneli, Modood and Bradley, 2005; Modood et al., 1997).

### Step 1. I-positions: From what I-positions does the Self speak?

The aim of this step is to identify the various I-positions from which the participant speaks.

#### Code first-person pronouns

We first code first-person pronouns (singular and plural: I, we, me, us, mine, ours, myself, ourselves). It can be useful to include first-person possessive determiners such as ‘my’ and ‘our’. Where someone is speaking on behalf of an organisation, group or even nation, it will also be necessary to code organisation or group names that are the subject in the sentence.

The next step is to code all the utterances pertaining to these pronouns, possessives or groups. The boundaries of the coded segments in this step are determined by the shifts in perspective made by the speaker, so that each coded segment expresses a single I-position. The boundaries between different I-positions may sometimes be difficult to disentangle; in these cases it may be appropriate to mark (part of) an utterance as belonging to more than one I-position.

#### Group coded segments into distinct I-positions and summarise each

All the utterances expressing a single I-position can then be collated to reflect one voice, and labelled in the form ‘I-as-X’. It may be desirable to label (and characterise) all the I-positions identified; alternatively, iterative analysis of the data may lead to exclusion of some I-positions as irrelevant to the research question(s). Finally, it should be possible to characterise each I-position; for example, what are the salient features, views or values associated with it?

For example: below is a coded excerpt from Adem’s interview.

Emma: So when you go to Cyprus or Turkey- how do the family see you when you go?

Adem: Well my auntie, she knows that **I’m** more of a Turkish culture- that **I** am involved more in Turkish culture and language and stuff-

Emma: Than what?

Adem: like Culture- like if **I** was like to go out for a meal **I** would go out to a Turkish restaurant. If **I** was to, like special days they’ve got, **I** am always there. More involved with Turkish people. If **we** go out to a music night, **we** go out to a Turkish concert, stuff like that.

Emma: With your friends from here or friends from school?

Adem: From school, outside school, mixed- and also there are some people out in Cyprus who think **I’m** English or German because **I’m** so white- most of them are brown, dark skinned and **I** said ‘no **I’m** not’.

Emma: How does that make you feel when they say, ‘oh, you’re English’.

Adem: It doesn’t make **me** feel bad- but **I** just tell them that **I’m** not- basically, but *it doesn’t bother* ***me*** *really because* ***we*** *are all one, everyone is a people like, everyone’s one really.* ***I*** *don’t discriminate or say that he is different race or he is from there*, ***I*** *just see everyone as one.*

Following the sub-steps outlined above, we coded first-person pronouns (marked in bold), and then grouped the related utterances into two voices speaking from two distinct I-positions (marked with underlining or italics). The most prominent voice in these excerpts is Adem’s ‘I-as-Turkish’ voice (underlined). We identified his ‘I-as-Turkish’ voice by looking at what group(s) the ‘we’ and the ‘I’ belong to (e.g. ‘we’ Turkish people who go out to Turkish places), by considering the views, values, claims or attitudes being expressed (e.g. ‘I’m more of a Turkish culture’) and examining the contrasts with who or what the ‘I’ claims *not* to be (e.g. ‘I’m not English’).

In the latter part of the excerpt, there is a shift to a second I-position, which we labelled ‘I-as-human’ (marked in italics). This shift to a different voice – marked by a ‘but’ – is distinguished by a change in perspective, from one focused on exclusive English or Turkish identities to an inclusive one that argues race is not relevant; according to I-as-human, ‘we are all one’.

In many cases, the surrounding text is insufficient to fully determine the I-position from the first person pronouns. For example, when the interviewer tries to clarify who Adem goes out with, another salient characteristic of the ‘we’ could arguably be their age. This kind of ambiguity can often be resolved through an iterative approach to analysis. In this instance, Adem’s I-as-Turkish position, and its dominance, became clearer when examined in the context of the complete interview, findings from step two, his focus group and supplementary data. This highlights the importance of coding in the context of the whole, and not segmenting and de-contextualising utterances.

Once all utterances associated with an I-position were coded, we could characterise the voice. For example, Adem’s I-as-Turkish voice is associated with Turkish friends and family, emphasises the importance of involvement in Turkish culture, and espouses a discourse of ethnocultural purity, suggesting one cannot identify as both Turkish *and* English.

### Step 2. Voices of inner-Others: What other voices can be heard?

Inner-Others are rarely physically present (as the interviewer is); rather they are given voice within and alongside the ‘I’, shaping and colouring the meaning of utterances. Since inner-Other voices might belong to other individuals or groups, or reflect discourses and social languages, the origin of voices can be difficult to identify and will likely require an iterative approach and interpretive work that references supplementary sources.

Several different techniques are needed to identify the voices of inner-Others. We list them here, beginning with those that require least interpretive work and ending with those requiring more sensitivity to the wider context.

#### Code all third-person pronouns and named individuals or groups

Similar to step one, we begin by coding uses of third-person pronouns (he, her, them, etc.), third-person possessives (their, his, etc.), named individuals, and groups or organisations. For each, we code the surrounding text for which the pronoun/name is the referent. These coded segments can then be grouped into clusters and given a label, reflecting who the voice belongs to. This will map out the significant Others within the data, providing a kind of ‘reference list’ which may be useful when trying to identify the social origins of indirect quotes and echoes. For example, in the excerpt from Adem’s interview (above) we can identify several Others, including his auntie, friends from school and people in Cyprus.

#### Code all direct quotes

Direct quotes (a form of reported speech) are straightforward to identify and usually have a clear attribution. For example, in this excerpt Mehmet quotes directly from Atatürk’s ‘pledge’, ‘*Andimiz*’:

Mehmet: It’s like a speech said by **Atatürk** which was, *it just signifies that you are Turkish- what you should- its’ just like a basic law saying you have got to do anything you can to preserve Turkey or Cyprus, whatever, and like at the end they say, ‘Ne mutlu turk’um diyene!’ That’s like, ‘Forever be happy that you’re Turkish’ so it just signifies that you should be proud of who you are.*

#### Code all indirect quotes

Indirect quotes are a form of reported speech which does not include precise quotation; often the person or group to whom an indirect quotation is attributed is also less precise. For example, when Adem talks about his visits to Cyprus he uses indirect quotes:

Adem: there are **some people out in Cyprus** *who think I’m English or German because I’m so white.*

The indirectly quoted voice here belongs to a vaguely defined group rather than an individual – ‘some people out in Cyprus’. This generalised Other constitutes an inner-Other in Adem’s dialogical Self whose voice (telling Adem he seems English) appears in his autodialogue.

#### Code all ‘echoes’

In echoes and ‘ventriloquations’, there is no explicit reference to the voice of an Other (as in reported speech), but there are nonetheless indications that the utterance has a distinct social origin beyond the speaker. In some instances, the researcher may be alerted to a possible echo by words that sound ‘foreign in the mouth’ of the speaker (Bakhtin, 1981: 293). Another approach to identifying echoes is to triangulate different sources of primary data (e.g. different participants) or to cross-reference with supplementary data (e.g. wider societal discourses). For example, through an iterative process of analysis, drawing on reported speech from across the data, notes from observations, and discourses about ethnicity described in the literature, we identified a generalised Turkish community voice that exhorted the young people in our sample to be proud of being Turkish and not forget their Turkish heritage. In some instances this voice appeared as reported speech associated with specific Others. For example:

Ahmet: I do sometimes [say my name the English way]- but well- the thing is like back in the day I did- but **my dad’s** taught me like *– you gotta show who you are*

A few lines later in the same interview, we hear this voice as an echo, albeit one that has been internalised:

Emma: And who are you?

Ahmet: Turkish-Cypriot and proud to say it, yeah

Reinforcing our interpretation that this is an echo, from other interviews we learned that this view does not just come from Ahmet’s father, but Turkish school teachers and, as Mehmet explained, Atatürk’s ‘pledge’ which was recited in the Turkish school assemblies.

Through triangulation and iterative readings of the data, we were able to identify echoes of this discourse of Turkish pride in utterances which were not obviously multivoiced on first reading.

#### Ask who an utterance is addressed to

With this final technique for identifying the voices of inner-Others, we switch from focusing on the *source* of an utterance or voice, to its *audience*. Drawing on the notion of ‘addressivity’ (Bakhtin, 1986), this technique means asking to whom, present or physically absent, the utterance is oriented (other than the researcher). For example, in one focus group, two boys rejected an English identity; another (Mehmet) commented that ‘we just don’t fit in in this country’; a fourth, Ahmet, then disagreed:

Ahmet: This [London/England] is my home- sorry boys but it is [laughter in the group] […] I mean- don’t get me wrong- I’m still Turkish- d’you know what I mean? Turkish and proud of it- it don’t mean that just cos- just cos- I fit in here-

Mehmet: Yeah yeah- that doesn’t make you a bad person just by saying that- it’s just an opinion, innit?

Ahmet is clearly addressing the other focus group participants (‘sorry boys’) when he defends his sense of belonging in England, just as Mehmet is addressing Ahmet when he says ‘you’. At the same time, however, Mehmet and Ahmet are simultaneously addressing (and thereby invoking) an Other who is not present: the generalised voice of their Turkish community who *might* say that ‘fitting in’ in England *does* make Ahmet a ‘bad person’, and perhaps no longer Turkish (otherwise why would Ahmet say ‘sorry’ and why would Mehmet exonerate Ahmet?).

#### Group coded segments into distinct inner-Other voices and summarise each

The utterances associated with different inner-Other voices can be grouped together (as in step one), given a label (indicating the individual or group from which it derives) and summarised (e.g. the salient views, values or tropes). For example, inner-Others identified in the excerpts above include: ‘people in Cyprus’ (who question these young people’s claims to a Turkish identity), parents’ voices and the generalised voice of the Turkish community (who exhort these young people to be proud Turks).

### Who is doing the talking?

Completing steps one and two allows us to answer the question ‘Who is doing the talking?’ (Wertsch, 1991). At this point it is useful to compile a table of all the voices that can be heard within the utterances of each speaker (i.e., for each transcript), along with a brief characterisation of each voice and excerpts illustrating this voice (see Table 1). This table will support step three, where the aim is to explore the interactions and interrelations between the different voices and their significance for the research question(s) at hand.

**Table 1.** Who is doing the talking? Sample table of voices compiled during steps one and two for Adem’s data set.^a^

**Internal I-positions**	*Characterisation*	*Illustrative quotes*
I-as-Turkish	Position reflexively claimed. Proud; knows Turkish heritage and language, involved in Turkish culture; loyal to Turkish community; discourse of ethnocultural purity (cannot be Turkish *and* English)	‘I am involved more in Turkish culture and language and stuff … if I was like to go out to eat, go out for a meal I would go out to a Turkish restaurant. If I was to, like special days they’ve got, I am always there.’
I-as-human	Expressed in talk but infrequently; inclusive view of society, everyone is ‘one’ by virtue of all being human	‘because we are all one, everyone is a people like, everyone’s one really’
**Inner-Other voices**		
Auntie in Cyprus	Supportive voice, recognises his Turkish identity, endorses/values his involvement in Turkish culture and knowledge of Turkish language. Part of wider Turkish community.	‘Well my auntie, she knows that I’m more of a Turkish culture- that I am involved more in Turkish culture and language and stuff’
Turkish community (generalised)	Generalised Turkish community. Emphasis on not forgetting Turkish identity and heritage; values loyalty to the group and involvement in Turkish culture/diaspora.	‘If we go out to a music night, we go out to a Turkish concert, stuff like that’
Some people in Cyprus	Voice of family or generalised other in Turkey or Cyprus – ‘some people’ (unclear boundaries). Sees children of diaspora as no longer Turkish because of ‘English’ values, attitudes, style or skin colour.	‘some people out in Cyprus who think I’m English or German because I’m so white- most of them are brown, dark skinned’

a This table, based on Adem’s interview, includes only those voices evident in the excerpts presented in this paper. The full interview contains more voices.

### Step 3. What are the interactions between voices in the Self?

There are three different types of interactions between Self’s voices to examine: 1) between specific I-positions and inner-Others; 2) between different I-positions; 3) between different inner-Others. There will be many possible permutations: which relationships a researcher wishes to pursue will depend on the research question(s), or how exploratory the analytic scope is. The sub-steps below aim to systematically draw out the interactions evident within the data:

1. Retrieve the instances of each I-position as they occur in the text, identify which other voices co-occur or immediately precede/succeed the I-position, and examine the autodialogue between the ‘I’ and those co-occurring voices.
2. Examine the relationship between the different I-positions and the content of their utterances; do this for all I-positions using the complete transcript (or all data for that individual), not just segments of the text where I-positions co-occur.
3. Referencing the table of identified voices, consider relations between any other voices not already examined (e.g. relations between different inner-Others) in the same way that relations between I-positions were examined.

Regardless of which relationships are examined in which order, for each relationship we want to understand the dialogical dynamics of those voices: are the voices contradictory, mutually reinforcing, supportive, questioning, etc.? Do autodialogue and interaction between the voices lead to resistance, reinforcement, silencing or transformation (Valsiner, 2002)? The questions below can be used to explore the nature of the relationships between the multiplicity of voices within the Self. This list is not exhaustive, but reflects our collective experience with empirical data and the literature synthesised by Gillespie and Cornish (in press).

#### Is there a relationship between these particular voices, and how close is it?

Some voices within the Self may stand in direct social relation to each other (e.g. participants’ I-as-Turkish position and inner-Others such as Turkish family in Cyprus), and are likely to appear in autodialogue with each other (e.g. ‘some people say I’m English … I say “I’m not”’). Other voices within the Self may co-exist in a state of symbiotic ambivalence (Valsiner, 2002), where there is little direct interaction and the existence of one may neither challenge nor reinforce the other.

#### How does one voice respond to other voices?

Examining autodialogue, consider how each voice (particularly the ‘I’) responds to other voices – does the response express a challenge, resistance, tension, hurt, endorsement, etc.? For example, Adem’s I-as-Turkish voice tries to counter the voice of ‘some people in Cyprus’ who ascribe him an English identity, indicating that the relationship between these two voices entails challenge and (more or less successful) resistance.

#### What are the evaluative overtones within the voice?

Here we consider the emotional or evaluative tone (Burkitt, 2010) of the voice, from the perspective of the ‘I’. For example, is the voice admonishing, praising, supporting or ridiculing? Returning to Adem’s interview, the voice of his aunt in Cyprus appears to be a supportive one that endorses his claims to an ‘I-as-Turkish’ position through approval of his involvement in Turkish culture.

#### Are there any ‘dialogical knots’ and how can these be explained?

‘Dialogical knots’ are points of conflict or tension within autodialogue. These are often indicated by words such as ‘but’ or ‘however’, or by a sudden switching from one voice to another, suggesting underlying tensions within the dialogical Self.

One such dialogical knot is indicated in the exchange between Ahmet and Mehmet reported above. Ahmet begins by saying ‘This [London/England] is my home’; he then feels the need to apologise for this (‘sorry boys’), before switching to the I-as-Turkish position to quickly reassert he is ‘Turkish and proud of it’. This switching of voices, the perceived need to apologise, and the idea that someone *might* ‘get him wrong’ are indicative of underlying tensions between an English identity position and a Turkish one. The switching in Adem’s excerpt – marked by a ‘but’ – from I-as-Turkish to I-as-human indicates a similar tension: while I-as-human claims ‘everyone is one’, the nature of the switch ‘but it doesn’t really bother me’ implies one *might* be ‘bothered’ if one were called ‘English’ and denied a Turkish identity by Turkish Others. Exploring these tensions allowed us to identify and explain key dynamics in these young Turks’ efforts to negotiate their ethnic identities in a complex, multicultural social field.

#### What are the power dynamics between the voices?

The dominance and power structure of one’s ‘real’ environment is reflected in dialogical relations within the ‘society of mind’ (Hermans, 2002), resulting in certain voices being ‘privileged’ or ‘silenced’, more or less temporarily (Wertsch, 1991). For example, while amongst our participants ‘I-as-Turkish’ was the dominant I-position, some participants (like Ahmet) also spoke at times from a more hybridised position, an ‘I’ that felt ‘at home’ in England. This position was much weaker, fraught with tension and often silenced by other voices within the Self, such as the Turkish community inner-Other which might frame the young person as ‘a bad person’.

### Bringing the analysis and research questions together

Finally, as with any analytic method, the results of the analysis are used to address the original research question(s). Depending on the nature of the question(s), an important part of this process may be comparing and contrasting the voices and dialogical dynamics identified in the talk/text of each participant. Thus far in our method we have described a process which is applied to each transcript (or dataset for a given participant); this is important, since this type of analysis is not concerned with comparisons across data sources of decontextualised utterances, but rather analysis of autodialogue and interactions between voices *within* individual Selves. This is not to say it is inappropriate to bring together, compare or synthesise data from different individuals, forming a group-level analysis. It is simply that this stage of the analysis comes after the talk/text from each individual (source) has been explored.

In our example paper, the multiplicity of voices and dialogical dynamics that were mapped out and characterised were brought to bear on the question of how identities are (co)constructed (Aveling and Gillespie, 2008). We described the different voices that populated the young Turks’ Selves at individual and group levels, and examined how their self-constructions were shaped by asymmetrical power relations between various socioculturally situated voices. We showed how second-generation Turks were caught in a tangle of loyalties and racialising discourses associated with the different communities of which they were part, and how these tensions manifested in the complex dialogical relations between the individual and collective voices that constitute the Self. Our analysis suggested that while the process of identity construction for this group was fraught with contradictory voices and unresolved dialogical struggles, the dynamic movement between I-positions nonetheless reflected an adaptive response to the power asymmetries that structured their sociocultural context.

## Discussion: principles and applications

In this paper we have sought to contribute a method for analysing qualitative data informed by the tradition of dialogism. Having presented the method, we now outline four principles to bear in mind when using the method, and indicate some of the areas of research to which this method could be profitably applied.

The four principles for the application of the method are informed by the epistemology of dialogism, and are cautionary in that adherence to these principles guards against reification of the method, that is, applying the method without regard for the particularities of the research question or context. First, analysis of multivoicedness cannot be done in isolation from context; insistence on the interdependence of Self, Other and the social field is essential to dialogism (Linell, 2009). Thus although we have presented the method in a series of categorical and linear steps, the application of the method must be sensitive to context and applied iteratively, rather than in a single pass over the data.

Second, fidelity to dialogism also implies a need to remain open to alternative interpretations. In common with other interpretive methods, certainty about an interpretation is never conclusive. For example, a researcher will never have all the possible information that may determine the origin of echoes. More radically, Grossen (2010) has argued that the attempt to develop methodological tools for the analysis of dialogue is incompatible with the assumptions of dialogism itself, and that applying a rigid method may simplify and ‘monologise’ complexity. Accordingly, our proposed three steps should be used with sensitivity, critical judgement and openness to alternative interpretations, not treated as a method producing a definitive answer.

Third, the method depends on significant interpretative skill and contextual knowledge. The method is therefore best applied where the researcher is able to triangulate the primary data with other forms of information about the wider social and symbolic context. Sources might include theoretical and sociological literature, mainstream and social media, cultural resources such as books, music and films, or indeed complementary primary (e.g. ethnographic) research. In the interest of scaffolding the development of such interpretative skills, Gillespie and Cornish (in press) outline a series of ‘sensitising questions’ that researchers can ask of data in order to inform a dialogical interpretation.

Fourth, reflexivity on the part of the researcher is crucial to the method. The researcher needs to be sensitive to how the research encounter itself may foreground particular voices and dialogical dynamics (Mertkan-Ozünlü, 2007). In the young Turks study, for example, we had to reflexively consider the potential influence of a white, British interviewer talking to second-generation Turkish youth in the context of their Turkish supplementary school. The method itself does in fact offer a systematic approach to being reflexive – as the voice of the researcher (and its appearance within the responses of participants) can be included in the voices and dynamics being analysed.

We now turn to considering the appropriate spheres of application of the method. While the method is suited to research questions about the interactions of Self and Others, it is distinctive in that it does not rely on data which records actual interactions between individuals or groups, nor on asking people to self-report on their interactions with the perspectives of others. Rather, the method analyses the interactions between Self and Other as they appear *within* the utterances of the multivoiced Self. As such it can be used in a wide variety of fields with most forms of qualitative data, including: individual and/or group interview data (e.g. Aveling, 2012; Aveling and Gillespie, 2008), documentary materials, ethnographic data involving a combination of forms of data (e.g. Gillespie, 2006), data from diaries (e.g. Gillespie et al., 2007) or biographical texts (e.g. Gillespie, 2005). This potential can be further expanded through the use of other forms of text pertaining to groups or organisations (e.g. policy documents, promotional material, meeting minutes) (Linell, 2009).

The method can also be applied to a wider set of research questions, beyond questions about the voices within the Self. According to dialogism, selves are co-constituted in micro- and macro-social relations. As such, analysing the multivoiced Self links the ‘micro’ to the ‘macro’ by examining the dialogical dynamics which structure knowledge, society and subjectivity *through* the ‘society of mind’. The method thus offers a way of approaching questions such as: how is social knowledge produced, and how is this shaped by the competing perspectives of groups and institutions and the power relations that sustain them? What are the social origins of the discourses through which individuals speak, and what does this window into the wider sociocultural context tell us? How are identities and subject positions (co)constructed, and how is this process mediated through interactions with Others in a particular symbolic context? Organisational research might investigate multivoicedness within organisations by examining, for example, the voices within an organisation’s documentation, mission statement, procedures and processes.

## Conclusion

The present article has contributed a method of analysis for analysing multivoicedness. The methodology is positioned between traditional analyses of discourse (i.e. conversation and discourse analysis) and more psychological analyses (i.e. questionnaires and self-report methods for documenting I-positions). The proposed method entails a close analysis of spoken or written text to identify the I-positions and Other-positions and the ways they interact. This method, we suggest, enables researchers to work at the intersection between discourse and psychology in a more systematic way. The success of any method is always in what it enables researchers to achieve (Cornish and Gillespie, 2009), and by this measure dialogism is already an important method. By contributing a rigorous and transparent procedure we hope to further enable research by, on the one hand, providing a guide for researchers unfamiliar with dialogism, and, on the other hand, giving researchers already familiar with dialogism a common framework and terminology for conceptualising research on multivoicedness.
